# Craniofacial Ciliopathies and the Interpretation of Hedgehog Signal Transduction

**DOI:** 10.1371/journal.pgen.1006460

**Published:** 2016-12-29

**Authors:** Karen J. Liu

**Affiliations:** Craniofacial Development & Stem Cell Biology, King’s College London, Guy’s Hospital, London, United Kingdom; University of Oxford, UNITED KINGDOM

An emerging body of literature has shown that cilia-dependent Hedgehog (HH) signaling is crucial to the patterning of the face. Ciliopathic mutations are frequently associated with craniofacial anomalies, and while the links are clear, the observed phenotypes can vary widely, leading to confusion about how these mutations affect processing of HH effectors. In November 2016’s issue of *PLOS Genetics*, Chang et al. uncover an important role for HH-dependent transcriptional repression during facial development.

## Cilia and Developmental Roles for HH Signaling

Cilia are tiny, hair-like projections found on the surface of eukaryotic cells. These projections can be motile, aiding in the movement of cells and surrounding liquids. There are also immotile cilia, which are notable in vertebrates for the interpretation of many extracellular signals [1]. Cilia are complex organelles, requiring a suite of proteins involved in cellular functions as disparate as protein synthesis, microtubule organization, vesicular trafficking, and intraflagellar transport. Therefore, it is no surprise that numerous studies in animal models have demonstrated that cilia proteins are crucial for the patterning of developing organs. Because the majority of cells have cilia, affected organ systems are diverse, and altered function could potentially lead to a wide range of human diseases. Recent advances in genetic analysis confirm this theory, as multiple mutations affecting cilium structure and function have been implicated in congenital anomalies. The craniofacial complex is one of the systems most commonly affected by cilia dysfunction.

Of the many external cues, HH signal transduction has emerged as a key molecular pathway reliant on functional cilia [2,3]. However, despite the many ciliopathic animal models that now exist, a great deal of confusion arises when studying the consequences of cilia mutations on HH. In some studies, mutations mimic a loss of HH signaling, while in other contexts, HH signaling appears to increase. This has been particularly evident in craniofacial structures, where loss of cilia can lead to both narrowing of the head with failure of separation of the forebrain hemispheres (holoprosencephaly, associated with loss of HH) and widening of the mid-face (associated with gain of HH) [4,5]. Altogether, this suggests that the phenotypic interpretation of HH signaling changes is more complicated than a simple on/off mechanism. In this issue, Chang et al. mutate the intraflagellar transport proteins *Ift88* and *Kif3a* in the mouse neural crest, comparing the phenotypes to those associated with functional mutations in the HH effectors *Gli2* and *Gli3* [6]. In doing so, they clarify the molecular basis for facial widening in ciliopathies and uncover a novel in vivo requirement for GLI-mediated transcriptional repression.

## Cilia and the Balance of GLI Functions

**Fig 1:**
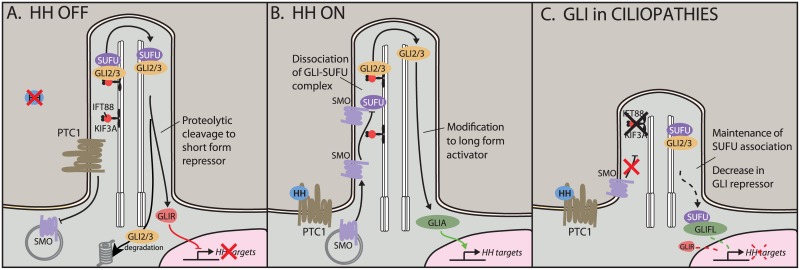
Hedgehog (HH) signaling in the cilium. A) In the off state, the HH receptor Patched 1 (PTC1) represses Smoothened (SMO), keeping it out of the cilium. The HH effectors GLI2 and GLI3 localize to the cilium with the help of transport proteins KIF3A and IFT88. Here, they can be proteolytically cleaved to short repressor forms. Full-length GLIs are targeted for degradation. B) In the presence of HH ligand, SMO translocates to the cilium, where it antagonizes the SUFU–GLI association, leading to production and nuclear translocation of GLI activators. C) In ciliopathic *Kif3a* and *Ift88* mutants, HH signaling is unable to disrupt the SUFU–GLI association. This shifts the nuclear ratio of GLIA:GLIR, leading to an increase in full-length GLI (GLIFL) and a reduction in levels of the shorter GLIR.

In vertebrates, the intracellular components of the HH pathway are sequestered in the cilium (Fig 1) [1]. Upon binding of the HH ligand to its receptor Patched (PTC), the transmembrane receptor Smoothened (SMO) translocates to the cilium, where it triggers the processing of the downstream transcriptional effectors GLI2 and GLI3 (Fig 1B). (A third GLI, GLI1, lacks a repressor domain and appears dispensable for embryonic development.) In the cilium, GLI2 and GLI3 associate with Suppressor of Fused (SUFU), and a SMO-triggered release of SUFU is necessary for subsequent GLI activator (GLIA) function. Both GLI2 and GLI3 can be modified to a long activator form or proteolytically cleaved to a truncated repressor form (GLIR). In vitro, both GLI2R and GLI3R are able to inhibit HH target genes; however, to date, the majority of evidence has suggested that GLI3 is the primary repressor in vivo, while GLI2 mediates the bulk of HH activation. In vivo, the difficulty in understanding HH signaling lies in the unique and overlapping functions of the GLI proteins. Until this report, an in vivo role for proteolytic processing of GLI2 had been elusive, particularly as previous reports demonstrated that the constitutively active *Gli1* could substitute for *Gli2* in a genetic knock-in [7].

Our current understanding of GLI processing in the cilium suggested that recruitment of SMO to the cilium leads to an increase in the ratio of GLI activator to GLI repressor (GLIA:GLIR) [8,9]. Therefore, tissue-specific interpretation of the GLIA:GLIR ratio could be due to different combinations of binding partners or varying accessibility of target promoters. Furthermore, we could postulate a dedicated set of GLIR targets that are entirely immune to GLIA binding and vice versa. Crucially, different tissues appear to use different ratios of GLIA:GLIR. Together, this suggests a complex interplay of three classes of GLI target genes. For example, HH signaling in dorsal–ventral patterning of the neural tube appears to balance GLI3 repression in the dorsal domain with GLI2 activation in the ventral domain [10]. In contrast, loss of GLI3R function in rhombomere 1 in the hindbrain is sufficient to de-repress expression of *fibroblast growth factor-8 (Fgf8)* [11]. Similarly, in the limbs, loss of GLI3R leads to severe polydactyly; here, the role of the ligand appears to be to limit the repressor, as loss of *sonic hedgehog (Shh)* skews the ratio toward GLI3R, resulting in a single digit forming [12,13].

In this paper, Chang et al. demonstrate that disruption of cilia function leads to an increase in the amount of full-length GLI2 and GLI3 in the nucleus, thus shifting the ratio of GLIA:GLIR toward the activator form [6]. This was partially rectified by increasing the amounts of GLI3R. However, complete genetic loss of all *Gli3* in the neural crest did not elicit the same phenotype, suggesting that GLI3 repressor did not encompass the full extent of the HH readout. Indeed, the midfacial phenotype was only seen when both *Gli2* and *Gli3* were eliminated, demonstrating that both GLIs contributed to the phenotype and raising the possibility of a compensatory role for GLI2R that is only uncovered in the absence of GLI3R. By systematically comparing a suite of ciliopathy mutations with different functional mutants of *Gli2* and *Gli3*, Chang et al. provide novel structure–function associations with distinct pathologies.

This paper also highlights the importance of tissue specificity in the interpretation of developmental phenotypes, especially with cues such as HH that are used reiteratively. By limiting mutations to the neural crest tissues, the authors were able to determine that a key function for HH in the neural crest mesenchyme is controlling the levels of transcriptional repression. Future experiments should examine more lineage-restricted tissues in the face as well as temporal requirements.

Of course, a number of open questions remain: Is the continued association with SUFU truly preventing GLI activator function? Is there a role for the cilium in GLI interactions with other regulators or post-translational modifiers? We also need a more accurate way to follow changing GLIA:GLIR ratios in vivo and to compare these to GLI-dependent responses. Finally, we must note that cilia are likely to coordinate multiple other signaling pathways; in the long term, how these pathways intersect will be crucial to our understanding of cilia and human disease.
